# Early Detection of Pepper Wilt Disease Using Unmanned Aerial Vehicle-Based Multispectral Time-Series Imagery

**DOI:** 10.3390/s26196119

**Published:** 2026-09-26

**Authors:** Gangin Je, Yeseong Kang, Chanseok Ryu, Changhyeok Park, Hojun Kwon, Dohyun An, Jian Kim, Sihyeong Jang

**Affiliations:** 1 Department of Bio-System Engineering, Institute of Agriculture & Life Science, Gyeongsang National University, Jinju 52828, Republic of Korea; jki6217@naver.com (G.J.); ryucs@gnu.ac.kr (C.R.); chang0140@naver.com (C.P.); smko0622@naver.com (H.K.); 2 Department of Smart Agro-Industry, Institute of Agriculture & Life Science, Gyeongsang National University, Jinju 52725, Republic of Korea; hwan7181@gnu.ac.kr (D.A.); mion0919@gnu.ac.kr (J.K.); 3 Fruit Research Division, National Institute of Horticultural & Herbal Science, Wanju 55365, Republic of Korea; jangsh6968@korea.kr

**Keywords:** disease, early detection, machine learning, multispectral images, pepper, unmanned aerial vehicle

## Abstract

This study evaluates early detection of pepper wilt disease using vegetation indices obtained from unmanned aerial vehicle (UAV)-based multispectral time-series imagery and machine learning classification. Multispectral images were acquired from June–August 2023, and disease status was determined by field diagnosis and visual interpretation of the imagery using diagnosed plants as references. Nine vegetation indices were used to develop k-nearest neighbors (KNN), support vector machine, and logistic regression (LR) models. For the June and July datasets, the best model was LR, achieving validation accuracies of 0.860 and 0.925, recall of 0.850 and 1.000, and F1-scores of 0.850 and 0.930, respectively. The best combined-dataset model was KNN; however, it did not outperform the monthly dataset models. The difference vegetation index showed the highest variable importance in the two monthly dataset models, and the variable importance of the normalized difference vegetation index was markedly higher in the July model. Similar trends were observed in the histogram analysis of vegetation indices and spectral features. Thus, date-specific models reflecting crop growth and disease progression were more effective than pooled observations across dates. Overall, UAV-based multispectral time-series imagery provides a practical basis for the field-scale early detection and prompt management of pepper wilt disease.

## 1. Introduction

Pepper (*Capsicum annuum* L.) is a vegetable essential for seasoning in Korean cuisine and accounts for large proportions of the total cultivation area and production of vegetables in Korea [1]. However, cultivated pepper is affected by various diseases, including powdery mildew, leaf blight, bacterial spot, and wilt disease. Wilt disease in pepper is commonly associated with soilborne vascular pathogens, particularly *Fusarium* spp. Infected plants generally exhibit leaf-yellowing, wilting, growth retardation, browning or necrosis of roots and stem vascular tissues, and—in severe cases—death [2,3]. The external symptoms of wilt disease, which can spread readily within fields, often become apparent only after the pathogen has affected the root or vascular system, and the resulting delay in response can considerably affect yield and quality. Therefore, early detection and effective management are essential and constitute an important area of study.

In recent years, remote sensing using unmanned aerial vehicles (UAVs) has been increasingly used in agricultural fields for crop-growth assessment and disease detection, mapping, and monitoring. Unlike conventional field surveys, UAV imagery enables the rapid, nondestructive acquisition of high-resolution spatial information, reducing labor and time required for crop and disease monitoring [4]. Among UAV-mounted sensors, multispectral sensors provide more information by utilizing a broader wavelength range than RGB sensors. In addition, the processing of multispectral data can be performed more efficiently and cost effectively than hyperspectral-data processing. Multispectral imagery is useful for detecting disease-induced changes in crop canopies because it can capture changes in visible, red edge (RE), and near-infrared (NIR) reflectance associated with canopy vigor, chlorophyll status, and physiological stress [4]. However, reflectance values extracted from multispectral imagery are sensitive to environmental conditions such as changes in illumination caused by sunlight and shadows. These effects can be mitigated using vegetation indices derived by combining two or more spectral bands.

Previous studies using multispectral imagery for crop disease detection have investigated grapevine fungal disease [5], banana wilt disease [6], and early wilt disease in pine trees [7]. In addition, researchers have used machine learning (ML) classification algorithms to detect diseases in rice leaves [8] and maize leaves [9]. Recent field-based studies have further demonstrated the applicability of UAV-based multispectral imagery in crop disease monitoring. Blasch et al. [10] used UAV-based multispectral imagery to monitor wheat rust and reported that spectral features useful for disease assessment varied across crop-growth stages. Radócz et al. [11] evaluated vegetation indices derived from UAV-based multispectral imagery for identifying maize smut under field conditions. In addition, Tuğrul et al. [12] demonstrated the early detection of Cercospora leaf spot and powdery mildew in sugar beet using UAV remote-sensing time series and ML before visible symptoms appeared. These studies indicate that UAV-derived spectral information can support field-scale disease detection and that the timing of data acquisition can influence disease-related spectral responses. Many studies on crop disease detection have used RGB-based image datasets collected under laboratory conditions [13,14,15]. Previous studies have reported on pathogenic agents and symptoms that are characteristic of pepper wilt disease [2,3]. Image-based deep learning models have also been developed to detect bell pepper leaf disease using leaf imagery obtained in the visible-light region [16]. However, field-based studies evaluating whether UAV-derived multispectral information acquired before subsequent disease confirmation can support the early detection of pepper wilt disease remain limited.

Therefore, this study aimed to evaluate the feasibility of early detection of pepper wilt disease using UAV-based multispectral time-series imagery and ML classification. The main contribution of this study is the field-based temporal disease assessment, which links spectral information acquired during an earlier UAV observation to infection status determined during subsequent assessment. Date-specific models for June and July were compared with a model trained on the combined dataset to examine how image acquisition timing and disease development stage affect spectral separability and classification performance.

## 2. Materials and Methods

### 2.1. Study Site and Plants

This study was conducted in a farmer’s pepper (*C. annuum* L., Colormura) field in Yechon-ri, Yongji-myeon, Gimje-si, Jeollabuk-do, Republic of Korea (Figure 1a). An aerial image of the field is presented in Figure 1. Seedlings were transplanted on 2 May 2023, and 30 plants were selected for the monitoring and field diagnosis of wilt disease. The 1991–2020 climate normals for the nearby Jeonju Branch Office of Meteorology were obtained from the Korea Meteorological Administration. The Jeonju Branch Office is a long-term meteorological station located near the study site [17]. The data show that the annual mean temperature and annual precipitation in the area are 13.7 °C and 1299.3 mm, respectively. Based on the Rural Development Administration’s soil characteristics map in the Korean Soil Information System (Heuktoram) database, the surface soil at the study site has a loamy texture [18].

### 2.2. Multispectral Image Acquisition

Multispectral images were acquired using a Matrice 300 RTK rotary-wing UAV (DJI Technology Inc., Shenzhen, China) equipped with an Altum-PT multispectral sensor (MicaSense Inc., Seattle, WA, USA) (Table 1). Flights were performed at an altitude of 25 m and a speed of 4 m/s. The forward and side overlaps were both set to 75%. Image acquisition was performed at solar noon on 28 June, 27 July, and 26 August 2023, to minimize shadow effects and ensure consistent illumination conditions.

### 2.3. Calculation of Vegetation Indices

The acquired images were mosaicked using Pix4Dmapper Pro 3.1.22 (Pix4D S.A., Prilly, Switzerland), pansharpened, and then registered in QGIS 3.28.11 (QGIS Development Team, Grüt, Switzerland). Vegetation and nonvegetation areas were separated using the excess green (ExG) index in ENVI 5.3 (Exelis Visual Information Solutions Inc., Boulder, CO, USA), and reflectance values were extracted for each plant (Figure 1c). The ExG index is defined as follows:

ExG = 2 × Green − Blue − Red.(1)

Because reflectance values are sensitive to environmental variations, vegetation indices were calculated to reduce variability and enhance spectral information. Nine vegetation indices were used, namely normalized indices (normalized difference vegetation index [NDVI], green NDVI [GNDVI], normalized difference red edge [NDRE], and normalized blue–green difference index [NBGVI]), simple ratio indices (ratio vegetation index [RVI], green RVI [GRVI], and difference vegetation index [DVI]), and modified indices (optimized soil-adjusted vegetation index [OSAVI] and transformed chlorophyll absorption in reflectance index [TCARI]) (Table 2).

### 2.4. Data Collection and Dataset Construction

The 30 monitored plants were analyzed for wilt disease infection (Figure 2a). During field surveys, a plant disease expert diagnosed 15 plants as infected on 27 July 2023, and an additional 11 plants were diagnosed as infected on 25 August. These field-survey data were used as reference information for determining the occurrence of wilt disease infection (Figure 2c).

However, the number of field-survey samples was inadequate for developing ML classification models. Therefore, additional samples were obtained from the same field through the visual interpretation of temporal canopy changes across UAV images acquired on 28 June, 27 July, and 26 August 2023 (Figure 2b). The plants diagnosed as infected during the field surveys served as visual reference samples for image-based labeling (Figure 2d). The canopy conditions and temporal changes at other plant locations were compared with those of the field-diagnosed plants. For image-based labeling, only plants showing clear and complete death in the subsequent UAV imagery acquired after approximately one month were classified as infected (Figure 2e), whereas plants that maintained stable canopy conditions without clear wilt-related decline throughout the observation period were classified as noninfected. Image-based labeling was performed solely through the visual interpretation of temporal canopy changes, and spectral reflectance or vegetation indices were not used as labeling criteria.

Following image-based labeling, a spectral consistency assessment was conducted separately for the June and July datasets to examine whether the samples designated as infected in image-based labeling exhibited spectral characteristics comparable to those of the field-diagnosed infected samples. As this assessment was performed after labeling, it was not used to assign, exclude, or modify infection labels. The original five-band reflectance values of the two sample sources assigned before interquartile range (IQR)-based outlier removal were compared using mean spectral profiles (Figure S1) and Welch’s two-sample *t*-tests. No significant differences were observed for four of the five spectral bands in June or for any of the five bands in July, with only the June NIR reflectance values showing significant differences (*p* = 0.014; Table S1). Source separability was also evaluated using k-nearest neighbors (KNN), support vector machine (SVM), and logistic regression (LR) classifiers under stratified five-fold cross-validation, with random oversampling applied only to the training folds. The mean balanced accuracy was 0.511–0.622 in June and 0.458–0.606 in July. Meanwhile, the mean area under the curve (AUC) for the receiver operating characteristic (ROC) curve was 0.492–0.681 in June and 0.404–0.669 in July (Table S2 and Figure S2). Overall, no strong or consistent spectral separation was observed between the field-diagnosed infected samples and those marked as infected in image-based labeling.

Based on the subsequently determined infection status, two analytical datasets were assembled by pairing vegetation indices from each UAV acquisition date with the corresponding subsequent infection-status labels. For the June dataset, vegetation indices derived from the UAV-based multispectral imagery, acquired on 28 June 2023, were used as predictor variables, and the infection status, determined approximately one month later on 27 July 2023, was used as the class label. For the July dataset, vegetation indices derived from the UAV-based multispectral imagery, acquired on 27 July 2023, were used as predictor variables. Further, the infection status was subsequently determined through field assessment, conducted on 25 August 2023, for the monitored plants and using the UAV imagery, acquired on 26 August 2023, for the image-labeled samples. Thus, both monthly dataset models evaluated whether spectral information acquired at an earlier observation date could distinguish plants according to their subsequently determined infection status. The temporal relationship between UAV image acquisition and the subsequent determination of infection status for the June and July datasets is summarized in Figure 2f. To visualize temporal canopy changes and spectral differences, representative UAV image sequences of two field-monitored plants, one remaining noninfected and one newly diagnosed as infected during the 25 August field assessment, were compared across images acquired on 28 June, 27 July, and 26 August. In addition, the distributions of NIR reflectance, DVI, NDRE, and NDVI in the June and July datasets were visualized according to the corresponding infection-status labels determined subsequently (Figure S3).

Finally, the June and July datasets were integrated into a combined dataset, which was used to evaluate the overall applicability of the proposed classification approach across different image acquisition dates and disease-progression stages. The number of samples in each dataset is summarized in Table 3.

### 2.5. Statistical Analysis

Statistical analyses were conducted using Jupyter Notebook 6.3.0 (Python 3.8.8, Project Jupyter, USA). Outlier reflectance values were removed using the IQR method [26]. Three ML classifiers were employed: KNN [27], SVM [28], and LR [29].

All classifiers were implemented using scikit-learn version 1.1.1. The dataset was for a single-year and a single field, the sample size was limited, and the primary objective was to compare disease-detection performance across acquisition dates, data-split ratios, and classifiers under consistent analytical conditions. Hence, extensive hyperparameter optimization was not performed. Instead, a fixed baseline configuration was specified for each classifier and applied across all acquisition dates and data-split ratios to allow standardized comparisons and computational reproducibility. The KNN classifier used five nearest neighbors with uniform weighting and Euclidean distance. Next, the SVM classifier used a radial basis function (RBF) kernel with C = 1.0, gamma = “scale,” and no class weighting. Finally, the LR classifier used L2 regularization with C = 1.0 and no class weighting, with the maximum number of iterations set to 1000. Parameters not explicitly specified were retained under the default settings of scikit-learn version 1.1.1. Similar approaches using software defaults or fixed baseline configurations have been reported in previous plant disease and vegetation health classification studies in which scikit-learn was used [30,31].

Stepwise variable selection was used to determine the optimal set of independent variables. The datasets were divided into calibration and validation sets with ratios of 8:2, 7:3, and 6:4. For the June and July datasets, noninfected and infected samples were separated by class and independently partitioned using the same split ratio and a fixed random seed of 543, after which the corresponding subsets were combined. This procedure maintained similar class proportions between the calibration and validation datasets. For the combined dataset, a plant-level group-aware splitting procedure was applied using the plant identifier (plant_id) to account for repeated observations of the same plants in June and July. All observations of the same plant were assigned exclusively to either the calibration or validation subset while maintaining class proportions as close as possible to those of the full dataset. Therefore, both subsets contained observations from June and July, but no plant identifier was shared between the calibration and validation subsets. A fixed random seed of 543 was used for reproducibility.

Because several vegetation indices were derived from overlapping spectral bands, potential multicollinearity among the variables selected for the LR models was assessed using the variance inflation factor (VIF). The VIF analysis was performed using only the calibration subset for each monthly dataset LR model. For sensitivity analysis, variables with VIF values of ≥10 were iteratively removed until all retained variables had VIF values of <10. Further, the performance of the refined LR models was compared with that of the original LR models.

Model performance was evaluated based on accuracy, precision, recall, and F1-score [32]. The performance was visualized using confusion matrices (CMs), precision–recall (PR) curves, and ROC curves. The PR curve is used to evaluate the proportion of true positives (TPs) in all predicted positives. The ROC curve is used to assess overall classification performance and is more influenced by the negative class [33]. Higher values of AUC indicate better model performance.

KNN classifiers and SVMs with RBF kernels do not provide directly comparable intrinsic measures of feature importance, and LR coefficients represent a model-specific measure. Hence, permutation feature importance was applied to all three classifiers to evaluate the contributions of variables to model predictions using a common model-agnostic criterion [34]. For each fitted model, the value of each selected variable in the validation dataset was randomly permuted 100 times while those of the other variables remained unchanged. Variable importance was quantified as the mean decrease in validation accuracy relative to the original model performance, with a greater decrease indicating a larger contribution of the variable. For the best-performing model of each dataset, the mean and standard deviation (SD) of the permutation feature importance values across the 100 permutations were calculated for all selected variables; the results are provided in Table S4.

The best-performing model was selected based on the highest validation accuracy (Accuracy_V). For cases with identical Accuracy_V values, the model with a higher validation recall (Recall_V) was selected, as minimizing the number of false negatives (FNs) is critical for disease management. When Accuracy_V and Recall_V were identical, the model with higher overall PR_V and ROC_V values was selected. The AUC values of the PR curves for the calibration and validation datasets are denoted as PR_C and PR_V, respectively, and those of the ROC curves are denoted as ROC_C and ROC_V, respectively. The performance metrics are defined as follows:

(2) $$\mathrm{Accuracy}=\frac{\mathrm{TP}+\mathrm{TN}}{\mathrm{TP}+\mathrm{TN}+\mathrm{FP}+\mathrm{FN}},$$

(3) $$\mathrm{Precision}=\frac{\mathrm{TP}}{\mathrm{TP}+\mathrm{FP}},$$

(4) $$\mathrm{Recall}=\frac{\mathrm{TP}}{\mathrm{TP}+\mathrm{FN}},$$

(5) $$F1\text{-}\mathrm{score}=\frac{2\times \mathrm{Precision}\times \mathrm{Recall}}{\mathrm{Precision}+\mathrm{Recall}},$$

where TN indicates true negative and FP indicates false positive.

## 3. Results

### 3.1. Classification Results for the June Dataset

The mean, SD, number of samples, and two-sample *t*-test results for the noninfected and infected plants in the June dataset are presented in Table 4.

The two-sample *t*-test results for the June dataset showed statistically significant differences between the infected and noninfected plants across all vegetation indices. Except for the NBGVI and TCARI, all vegetation indices of the noninfected plants significantly exceeded those of the infected plants.

Table 5 presents the results obtained by applying the classification models to the June dataset.

The LR model with the 8:2 calibration-to-validation set ratio that used the DVI, NDRE, GNDVI, RVI, TCARI, GRVI, NDVI, and NBGVI as independent variables was selected as the best model for the June dataset because it yielded the highest Accuracy_V and Recall_V values. This model achieved an accuracy of 0.732 (0.860), precision of 0.829 (0.850), recall of 0.671 (0.850), and F1-score of 0.699 (0.850) for the calibration (validation) dataset.

Variable importance analysis of this model indicated that the DVI was markedly more important (0.370) than the other variables, followed by the NDRE (0.100), GNDVI (0.088), and RVI (0.073). The complete permutation feature importance results for all the selected variables, including mean ± SD across 100 permutations, are provided in Table S4. The validation CM, PR curve, and ROC curve of this model are presented in Figure 3.

The validation CM showed three FNs (infected samples incorrectly predicted as noninfected). The AUC values of the PR and ROC curves were also high, with PR_C = 0.806, PR_V = 0.921, ROC_C = 0.827, and ROC_V = 0.926, indicating overall larger areas than those for KNN (PR_C = 0.847, PR_V = 0.751, ROC_C = 0.877, and ROC_V = 0.834) and SVM (PR_C = 0.778, PR_V = 0.821, ROC_C = 0.833, and ROC_V = 0.868).

### 3.2. Classification Results for the July Dataset

The mean, SD, number of samples, and *t*-test results for the noninfected and infected plants in the July dataset are presented in Table 6.

The *t*-test results for the July dataset showed statistically significant differences between the infected and noninfected plants across all vegetation indices except for the NBGVI. Except for the TCARI, all vegetation indices of the noninfected plants were significantly higher than those of the infected plants.

Table 7 presents the classification results obtained using the KNN, SVM, and LR models for the July dataset.

The SVM and LR models showed identical classification performance in the July dataset, with an accuracy of 0.834 (0.925), precision of 0.812 (0.870), recall of 0.873 (1.000), and F1-score of 0.841 (0.930) for the calibration (validation) dataset. Therefore, the optimal model was selected by comparing the AUC values of the PR and ROC curves. The SVM model showed PR_C = 0.914, PR_V = 0.983, ROC_C = 0.907, and ROC_V = 0.982, and the LR model showed PR_C = 0.915, PR_V = 0.988, ROC_C = 0.908, and ROC_V = 0.987. Overall, the LR model exhibited larger AUC values. Hence, the LR model with an 8:2 calibration-to-validation set ratio that used the DVI, NDVI, NBGVI, TCARI, RVI, NDRE, GRVI, and GNDVI as independent variables was selected as the best model for the July dataset.

Variable importance analysis of this model showed that the DVI (0.240) and NDVI (0.221) exhibited markedly higher importance than the other variables, followed by the NBGVI (0.062), TCARI (0.060), and RVI (0.045). The complete permutation feature importance results for all the selected variables, including mean ± SD across 100 permutations, are provided in Table S4. The validation CM, PR curve, and ROC curve of this model are presented in Figure 4.

For some models mentioned in Table 5 and Table 7, the validation accuracy was higher than the calibration accuracy. Because each calibration–validation partition was generated using a single, fixed hold-out split while maintaining similar class proportions, the calibration and validation subsets could still differ in their distributions of vegetation index values and degree of class separability. Hence, given the limited sample size, the higher validation accuracy was attributed to sampling variability in the hold-out partitioning rather than to systematically better performance of the model on validation data.

The validation CM showed zero FNs, indicating that all infected samples were successfully detected.

The VIF-based sensitivity analysis reduced the number of predictor variables of both monthly dataset LR models to four variables, namely DVI, NDRE, TCARI, and NBGVI, with all showing VIF values of <10 (Table S3). After removing highly collinear variables, the June model showed a validation accuracy of 0.837, recall of 0.800, and F1-score of 0.821. For comparison, the corresponding values were 0.860, 0.850, and 0.850, respectively, for the original model. In contrast, the refined July model retained the same validation accuracy (0.925), recall (1.000), and F1-score (0.930) as the original model.

The DVI was found to be the variable with the highest importance in the June and July models. The NDVI showed low importance (0.023) in the June model but substantially higher importance (0.221) in the July model. This result was further investigated by analyzing differences between the histograms of reflectance values and vegetation indices of the noninfected and infected plants.

### 3.3. Histogram Analysis of Reflectance Values and Vegetation Indices

The histograms of reflectance values and vegetation indices for the noninfected (green) and infected (red) plants in June and July are presented in Figure 5.

In June, differences between the noninfected and infected plants were clearer in the RE and NIR bands than in the blue, green, and red bands. Among the vegetation indices, the DVI, OSAVI, and TCARI showed distinct differences in the distribution of the two classes (noninfected and infected).

As in June, the RE and NIR bands showed clear differences between the noninfected and infected plants in July. However, compared with June, the separation between classes was clearer across all indices except for the NBGVI. In particular, the clearer separation of the classes in terms of the NDVI was consistent with the greater importance of the NDVI in the July model.

Overall, the histogram results showed that spectral separation between the infected and noninfected plants was more distinct in July than in June. These results highlight the better classification performance of the July model and indicate that disease-related spectral differences became clearer as the observation date approached the field diagnosis date.

Figure S3 shows these temporal changes. The field-monitored plant that remained noninfected maintained its canopy from the first to the third UAV image acquisition date, whereas the plant newly diagnosed as infected on 25 August showed marked canopy loss in the 26 August image (Figure S3a,b). The distributions of NIR reflectance, DVI, NDRE, and NDVI further illustrate the spectral differences between the subsequently defined noninfected and infected classes (Figure S3c,d). In particular, the NIR reflectance distribution of the infected class in the June dataset was shifted toward lower values than that of the noninfected class, consistent with Figure 5. Meanwhile, class separation based on the selected vegetation indices became more pronounced in the July dataset.

### 3.4. Classification Results for the Combined Dataset

The combined dataset was analyzed to determine whether increasing the number of samples by integrating data from different image acquisition dates improved model performance. In the plant-level group-aware splitting procedure, no plant identifiers overlapped between the calibration and validation subsets for any of the 8:2, 7:3, or 6:4 data-split ratios. The mean, SD, number of samples, and *t*-test results for the noninfected and infected plants in the combined dataset are shown in Table 8.

The *t*-test results for the combined dataset showed statistically significant differences between the infected and noninfected plants across all vegetation indices. Except for the NBGVI and TCARI values, all vegetation indices of the noninfected plants significantly exceeded those of the infected plants.

Table 9 presents the classification results obtained using the KNN, SVM, and LR models for the combined dataset.

For the combined dataset, the KNN model with an 8:2 calibration-to-validation set ratio that used the DVI, NDRE, OSAVI, NDVI, RVI, and NBGVI as independent variables was selected as the best model because it showed the highest Accuracy_V value. This model achieved an accuracy of 0.820, precision of 0.822, recall of 0.801, and F1-score of 0.812 during calibration and an accuracy of 0.815, precision of 0.833, recall of 0.769, and F1-score of 0.800 during validation.

Variable importance analysis of this model indicated that the OSAVI had the highest importance (0.179), followed by the DVI (0.162), NBGVI (0.161), and NDRE (0.106). The complete permutation feature importance results for all selected variables, including mean ± SD across 100 permutations, are provided in Table S4. The validation CM, PR curve, and ROC curve of this model are presented in Figure 6.

The validation CM showed nine FNs. The AUC values of the PR and ROC curves were as follows: for KNN, PR_C = 0.892, PR_V = 0.815, ROC_C = 0.916, and ROC_V = 0.855; for SVM, PR_C = 0.885, PR_V = 0.758, ROC_C = 0.873, and ROC_V = 0.799; and for LR, PR_C = 0.860, PR_V = 0.807, ROC_C = 0.858, and ROC_V = 0.814.

Although the combined dataset had a larger sample size than the monthly datasets, the validation performance of the model did not exceed that of the June or July models. In particular, the combined-dataset model showed lower Accuracy_V, Recall_V, and F1-score than the July model. Thus, simply increasing the sample size by integrating data from different observation dates did not necessarily improve the classification performance.

## 4. Discussion

This study demonstrates the feasibility of early detection of pepper wilt disease using vegetation indices derived from UAV-based multispectral imagery. The results indicate that UAV-derived multispectral vegetation indices can capture spectral differences associated with the subsequent occurrence of wilt disease in pepper. This finding is consistent with previous studies reporting that UAV-based multispectral imagery can be used to detect disease-induced changes in canopy reflectance values and vegetation indices [4]. In particular, studies on banana *Fusarium* wilt have shown that UAV-derived multispectral information, including RE and NIR spectral features, is effective for identifying wilt-infected plants in the field [6,35]. Although the analyzed crop species and diseases differ, these studies support the applicability of UAV-based multispectral imagery in the detection of wilt-related canopy stress in pepper.

In our previous study, the early detection of pepper wilt disease was evaluated using only plants whose infection status was determined through expert field surveys [36]. In that study, the best-performing model achieved a validation accuracy of 0.909, precision of 0.833, recall of 1.000, and F1-score of 0.909. However, the limited number of field-assessed plants restricted the size and representativeness of the dataset. In the present study, the expert-assessed plants were used as reference for interpreting time-series UAV imagery. Further, additional plants showing unambiguous wilt-related canopy changes were included in the dataset. The best model achieved a validation accuracy of 0.925, precision of 0.870, recall of 1.000, and F1-score of 0.930. Thus, compared with the previous study, the use of the expanded dataset resulted in a slightly higher validation accuracy, precision, and F1-score while the validation recall of 1.000 was maintained. These results suggest that expert-guided interpretation of subsequent UAV imagery may provide a practical means for supplementing limited field-survey samples for dataset construction. Spectral consistency assessment showed no strong or consistent spectral separation between the field-diagnosed and image-labeled infected samples (Figures S1 and S2, Tables S1 and S2). However, this assessment does not constitute independent pathological confirmation of the image-based labels. Because the two studies differ in dataset composition, model selection, and sample allocation, the observed performance differences should not be interpreted as resulting from dataset expansion.

A comparison between the June and July models indicated that the image acquisition timing strongly influenced the classification performance. The June model indicated the possibility of detecting plants that later developed wilt disease symptoms, suggesting that infection-related spectral differences may occur before field diagnosis. However, the July model outperformed the June model, indicating that the spectral separation between the infected and noninfected plants became clearer as the observation date approached the subsequent assessment date. This pattern is consistent with disease progression and associated changes in canopy conditions. The RE spectral region reflects changes in the chlorophyll content, biomass, and hydric status [37], and RE and NIR spectral features are useful for detecting *Fusarium* wilt and other crop diseases using UAV-based multispectral imagery [6,35].

The histogram and variable importance results further support this interpretation. In the June and July datasets, spectral differences between the infected and noninfected plants were clearer in the RE and NIR bands than in the visible bands. Moreover, the mean spectral differences between the noninfected and infected plants increased from June to July (DVI: from 0.022 to 0.052, NDRE: from 0.011 to 0.027, and NDVI: from 0.007 to 0.029), indicating greater spectral separation between the two classes in July. The DVI, which represents the difference between the NIR and red reflectance values, showed a high permutation feature importance in both monthly dataset models, suggesting that this spectral feature was consistently useful for distinguishing infected and noninfected plants. The NDRE also exhibited a greater between-class separation in July, consistent with increased disease-related differences in the RE band. In particular, the NDVI’s permutation feature importance increased markedly from 0.023 in June to 0.221 in July. This increase coincided with the larger difference in the NDVI between the two classes in July and indicated that the normalized contrast between the NIR and red reflectance values contributed more strongly to classification as the spectral separation between the infected and noninfected plants became more pronounced. Thus, these results provide a direct spectral basis for the higher classification performance of the July model and are consistent with previous studies reporting that vegetation indices can be used to assess crop disease severity [38,39].

The UAV imagery shown in Figure S3 provides visual context for the aforementioned interpretation: the field-monitored plants diagnosed as infected retained a canopy at earlier image acquisition dates but showed marked canopy loss in the 26 August image. Because the two classes in June and July were defined using different subsequent infection-assessment dates, the spectral distributions shown in Figure S3c,d should be interpreted as date-specific class comparisons rather than as a single temporal trajectory.

The LR models initially retained eight vegetation indices, several of which were derived from the same spectral bands and hence exhibited potential multicollinearity. The VIF-based sensitivity analysis reduced the number of predictor variables of both monthly dataset LR models to four variables (DVI, NDRE, TCARI, and NBGVI). The refined July model maintained the same validation performance as the original eight-variable model, whereas the refined June model showed a modest decrease in accuracy, recall, and F1-score. These results suggest that some of the correlated vegetation indices provided redundant predictive information, particularly in the case of the July model, for which the classification performance was unchanged after variable reduction. Therefore, although the overall classification pattern was maintained after removing highly collinear variables, the effects of individual predictors in the LR models should be interpreted cautiously because multicollinearity can affect the stability and independent interpretation of regression coefficients.

The combined dataset was analyzed to determine whether increasing the number of samples by integrating the June and July data could improve model performance. To avoid potential leakage caused by the use of repeated observations of the same plants made on different dates, all observations from each plant were assigned exclusively to either the calibration set or the validation set. Even after applying this plant-level separation, the combined-dataset model did not outperform the models trained on the monthly datasets. Thus, simply increasing the sample size did not necessarily improve the classification performance when data from different growth and disease development stages were pooled. The spectral characteristics of pepper canopies may vary depending on the crop-growth stage and disease progression. Therefore, integrating the June and July data may have increased within-class variability and introduced difficulties in learning consistent classification criteria. Similar temporal effects have been reported in recent UAV-based crop disease studies. For example, Blasch et al. [10] found that spectral features useful for wheat rust assessment differed across crop-growth stages, and Tuğrul et al. [12] highlighted the importance of time-series UAV observations for early disease detection in sugar beet. These findings, together with the results of the present study, indicate that the image acquisition timing and disease development stage should be considered when constructing disease-detection models using UAV-based multispectral imagery.

From a practical disease-management perspective, the June and July models provided different information for early detection and intervention. The June model was meaningful because it suggested the possibility of identifying plants that would develop wilt disease symptoms based on observations made earlier when spectral differences were still subtle. The July model, which exhibited the best validation performance and produced no FNs, was more suitable for minimizing missed detections. Because FNs can delay disease management and allow infected plants to remain in the field, recall is important in practical disease monitoring. Therefore, compared to using a simple combined dataset, constructing models according to the image acquisition date and disease development stage may be more appropriate for the early detection of pepper wilt disease.

Nevertheless, this study has several limitations. First, the experiment was conducted in a single field within the course of one growing season; therefore, additional validation across different regions, years, cultivars, and environmental conditions is required. Second, although additional samples were labeled based on field-diagnosed reference plants and subsequent UAV imagery interpretation, some uncertainty may have remained in image-based labeling. The supplementary spectral consistency assessment indicated no strong or consistent spectral separation between the field-diagnosed and image-labeled infected samples; however, this assessment cannot replace independent field- or laboratory-based pathological confirmation. Third, the UAV imagery was acquired at approximately monthly intervals, which limited the ability to observe continuous disease progression. Fourth, the classification analysis was limited to three conventional ML algorithms (KNN, SVM, and LR). These models were used for consistent and interpretable classification based on a single-year, single-field dataset rather than optimization of the predictive performance using increasingly complex architectures. Recent studies have demonstrated the potential of advanced ML and deep learning approaches for crop monitoring and disease detection using UAV-based multispectral information [40,41]. Therefore, future studies are required to evaluate such approaches using larger datasets that encompass multiple years, locations, cultivars, and environmental conditions to determine whether the performance gains can be generalized to different field conditions. Future studies should also incorporate more frequently acquired imagery, external validation datasets, and additional environmental or soil-related variables to improve the robustness and general applicability of artificial intelligence models used for the early detection of pepper diseases.

## 5. Conclusions

This study evaluated the feasibility of early detection of pepper wilt disease using vegetation indices derived from UAV-based multispectral imagery and ML classification algorithms. Spectral information acquired before field diagnosis could be used to distinguish between noninfected plants and plants that later exhibited wilt disease symptoms. A model trained on multispectral imagery acquired in June demonstrated potential for detection before disease confirmation. Meanwhile, a model trained on imagery acquired in July exhibited the highest classification performance and produced no FNs during validation. Thus, disease-related spectral differences became clearer as the observation date approached the diagnosis date. Histogram and variable importance analyses indicated that the separation between the infected and noninfected plants was mainly associated with RE and NIR reflectance characteristics. The DVI was consistently important in both monthly dataset models, whereas the NDVI was more important in the July model, suggesting that spectral differences related to vegetation vigor became more distinct as the disease progressed. The combined-dataset model did not outperform the monthly dataset models, indicating that simply increasing the sample size by integrating data from different image acquisition dates may not improve classification performance in cases involving various crop-growth and disease-progression stages. Therefore, compared with using a simple combined dataset, constructing models according to the image acquisition date and disease development stage may be more suitable for the early detection of pepper wilt disease. These findings demonstrate the potential of UAV-based multispectral imagery for field-based disease monitoring and provide useful information for developing precise strategies for managing diseases in cultivated pepper.

## Figures and Tables

**Figure 1:**
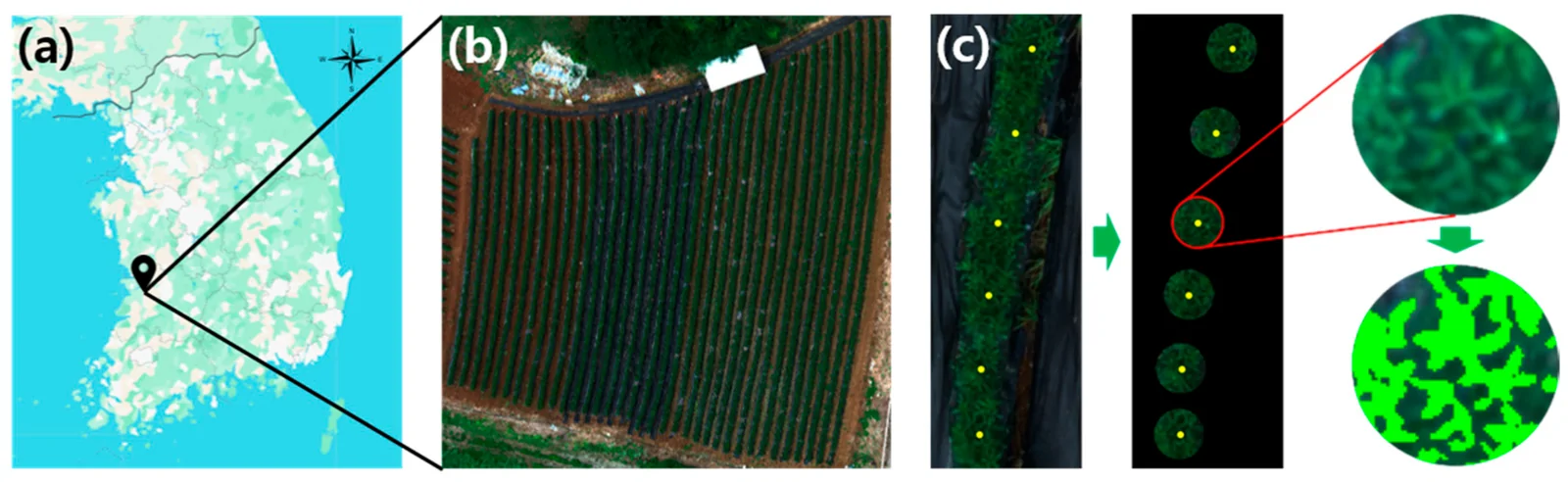
Location of study area and processing of imagery of individual plants: (**a**) location of the experimental field, (**b**) unmanned aerial vehicle (UAV)-based orthomosaic of the experimental field, and (**c**) region-of-interest selection and vegetation segmentation of individual pepper plants. Yellow dots indicate individual plant locations, and green arrows indicate the image-processing workflow.

**Figure 2:**
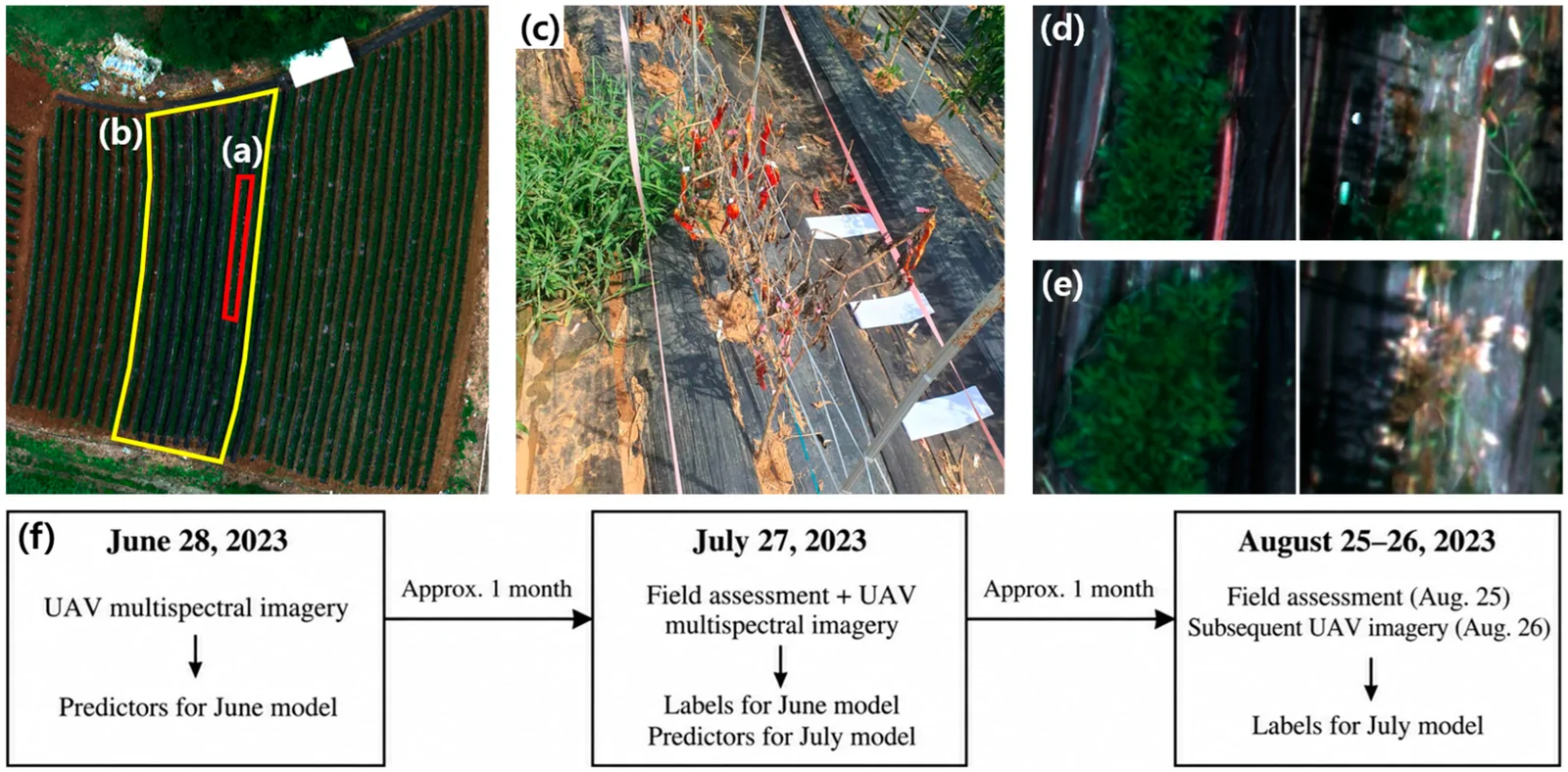
Sampling areas for (**a**) the field survey and (**b**) image-based labeling. (**c**) Ground-level photograph of an infected pepper plant. Earlier and subsequent unmanned aerial vehicle (UAV) images used for (**d**) the assessment of a plant during the expert field survey and (**e**) an additional plant whose infection status was determined through image-based labeling using expert-assessed plants as visual references. (**f**) Timeline showing the pairing of UAV image acquisition dates with subsequent infection-status assessment dates used to define the June and July analytical datasets.

**Figure 3:**
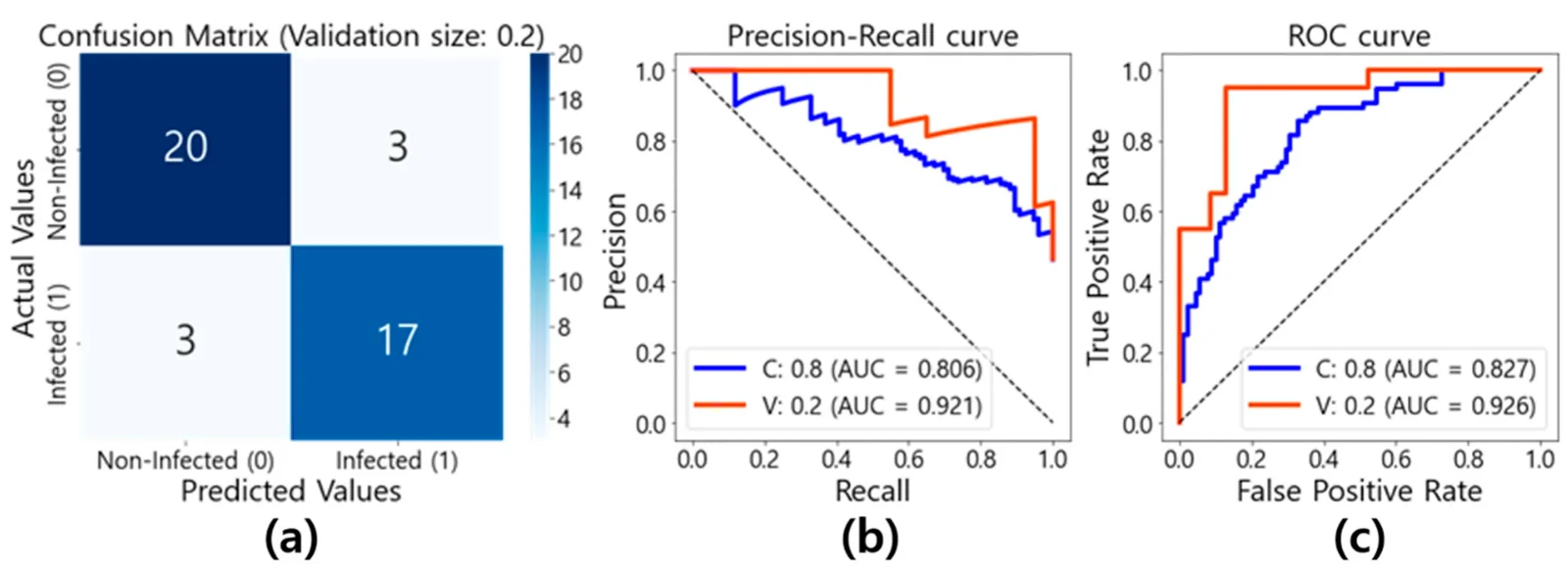
(**a**) Validation confusion matrix (CM), (**b**) precision–recall (PR) curve, and (**c**) receiver operating characteristic (ROC) curve of the logistic regression (LR) model for the June dataset. C, calibration; V, validation; AUC, area under the curve.

**Figure 4:**
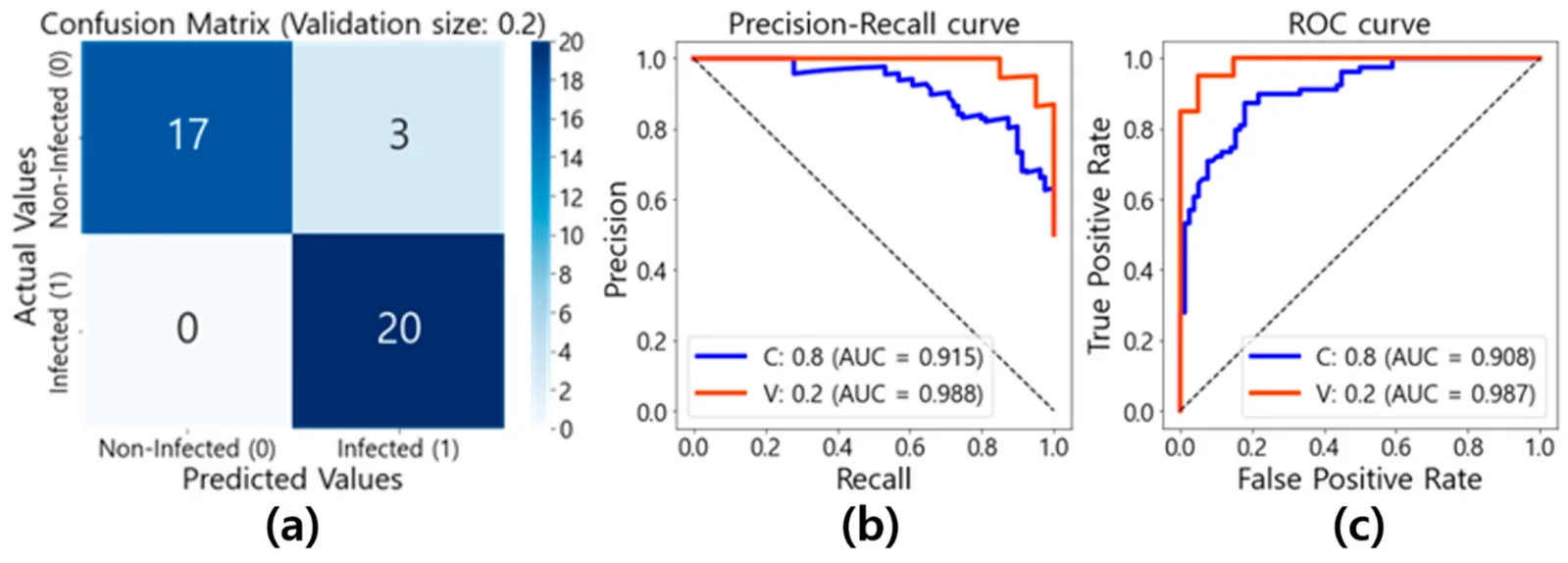
(**a**) Validation confusion matrix (CM), (**b**) precision–recall (PR) curve, and (**c**) receiver operating characteristic (ROC) curve of the logistic regression (LR) model for the July dataset. C, calibration; V, validation; AUC, area under the curve.

**Figure 5:**
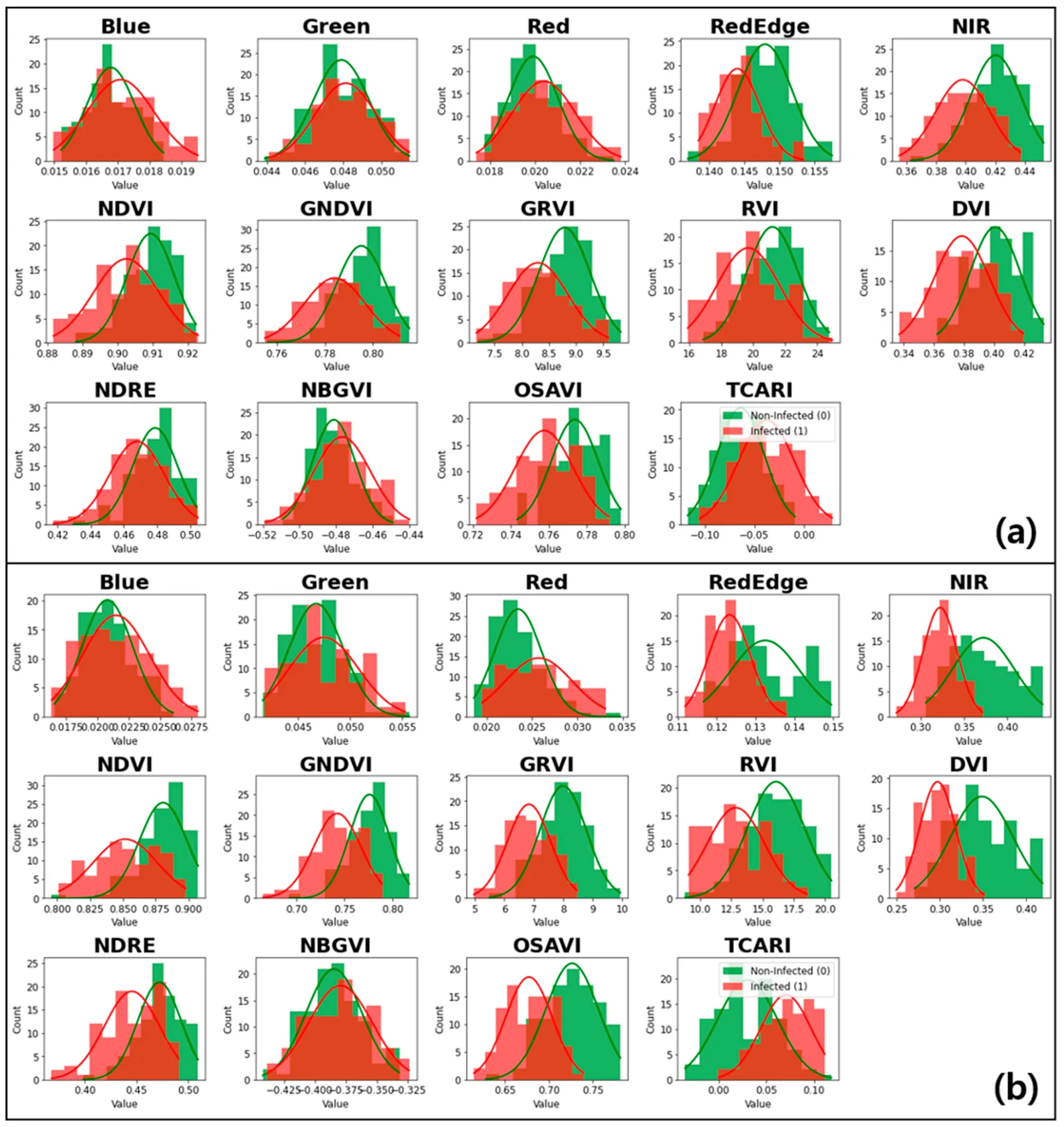
Histograms of reflectance values and vegetation indices in (**a**) June and (**b**) July. RE, red edge; NIR, near infrared; NDVI, normalized difference vegetation index; GNDVI, green normalized difference vegetation index; GRVI, green ratio vegetation index; RVI, ratio vegetation index; DVI, difference vegetation index; NDRE, normalized difference red edge; NBGVI, normalized blue–green difference index; OSAVI, optimized soil-adjusted vegetation index; TCARI, transformed chlorophyll absorption in reflectance index.

**Figure 6:**
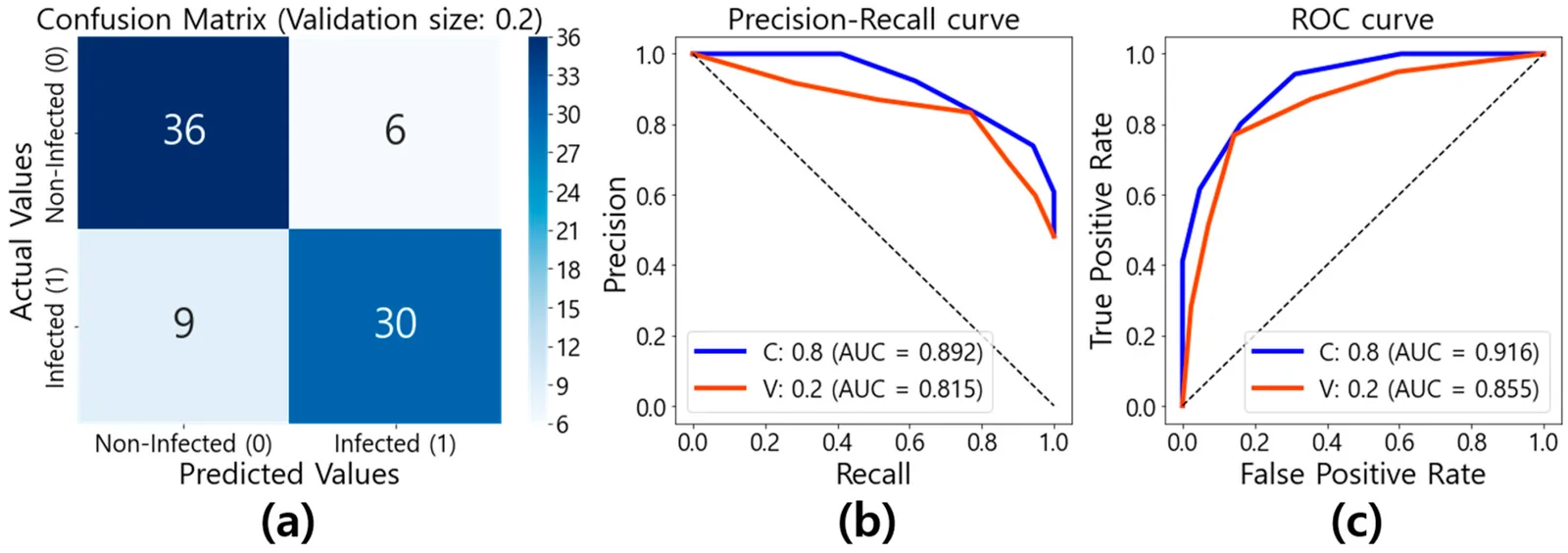
(**a**) Validation confusion matrix (CM), (**b**) precision–recall (PR) curve, and (**c**) receiver operating characteristic (ROC) curve of the k-nearest neighbors (KNN) model for the combined dataset. C, calibration; V, validation; AUC, area under the curve.

**Table 1:** Specifications of the unmanned aerial vehicle and multispectral sensor.

Category	Specification
**Unmanned aerial vehicle**	
Model	Matrice 300 RTK
Manufacturer	DJI Technology
Dimensions and weight	99.8 cm × 98.5 cm × 43.0 cm, 8.42 kg
Maximum flight time	~55 min
Maximum loadable weight	2.7 kg
Wind resistance	Up to 12 m/s
**Multispectral sensor**	
Model	Altum-PT
Manufacturer	MicaSense
Dimensions and weight	11.0 × 9.0 × 6.9 cm, 460 g
Center wavelengths and bandwidths	475 nm ± 32 nm (blue) 560 nm ± 27 nm (green) 668 nm ± 16 nm (red) 717 nm ± 12 nm (red edge) 842 nm ± 57 nm (near infrared) 634.5 nm ± 463 nm (panchromatic) 10.5 μm ± 6 μm (thermal)
GSD@25 m	1.08 cm/pixel
Panchromatic GSD@25 m	0.52 cm/pixel

**Table 2:** Vegetation indices used in the study.

Vegetation Index	Formula	Reference
NDVI	$\frac{\mathrm{NIR}-\mathrm{Red}}{\mathrm{NIR}+\mathrm{Red}}$	[19]
GNDVI	$\frac{\mathrm{NIR}-\mathrm{Green}}{\mathrm{NIR}+\mathrm{Green}}$	[20]
GRVI	$\frac{\mathrm{NIR}}{\mathrm{Green}}$	[21]
RVI	$\frac{\mathrm{NIR}}{\mathrm{Red}}$	[22]
DVI	NIR−Red	[22]
NDRE	$\frac{\mathrm{NIR}-\mathrm{RE}}{\mathrm{NIR}+\mathrm{RE}}$	[23]
NBGVI	$\frac{\mathrm{Blue}-\mathrm{Green}}{\mathrm{Blue}+\mathrm{Green}}$	[24]
OSAVI	$\frac{1.16(\mathrm{NIR}-\mathrm{Red})}{\mathrm{NIR}+\mathrm{Red}+0.16}$	[25]
TCARI	$3[(\mathrm{RE}-\mathrm{Red})-0.2\left(\mathrm{RE}-\mathrm{Green}\right)\left(\frac{\mathrm{RE}}{\mathrm{Red}}\right)]$	[25]

Notes: RE: red edge; NIR: near infrared.

**Table 3:** Number of samples in each dataset.

Dataset	Acquisition Date	Reference Date(s) for Infection-Status Determination	Noninfected	Infected	Total
June	28 June	27 July	114 (111)	102 (96)	216 (207)
July	27 July	25 or 26 August	108 (98)	105 (99)	213 (197)
Combined	-	-	222 (209)	207 (195)	429 (404)

Acquisition date: date of UAV image acquisition; reference date(s) for infection-status determination: date of field assessment or subsequent UAV imagery performed to determine the infection status. For the July dataset, field diagnosis of the monitored plants was conducted on 25 August 2023, and UAV imagery acquired on 26 August 2023, was used for image-based labeling. The numbers in parentheses indicate the sample sizes after outlier removal.

**Table 4:** Basic statistics of the June dataset after outlier removal.

Vegetation Index	Mean ± SD	
	Noninfected (111)	Infected (96)
NDVI	0.909 ± 0.007 a	0.902 ± 0.009 b
GNDVI	0.795 ± 0.010 a	0.784 ± 0.013 b
GRVI	8.787 ± 0.478 a	8.298 ± 0.554 b
RVI	21.20 ± 1.621 a	19.69 ± 1.965 b
DVI	0.401 ± 0.017 a	0.379 ± 0.019 b
NDRE	0.479 ± 0.014 a	0.468 ± 0.017 b
NBGVI	−0.481 ± 0.012 b	−0.477 ± 0.016 a
OSAVI	0.774 ± 0.012 a	0.758 ± 0.015 b
TCARI	−0.064 ± 0.024 b	−0.038 ± 0.028 a

Notes: SD: standard deviation; a, b: significantly different (*p* < 0.05). The numbers in parentheses indicate the sample sizes after outlier removal.

**Table 5:** Classification results of each model for the June dataset.

Model	KNN	SVM	LR
Data split	8:2	6:4	8:2
Selected variables	DVI, NBGVI, OSAVI, NDRE, TCARI, RVI, GRVI, and NDVI	DVI, TCARI, OSAVI, NBGVI, NDRE, and GRVI	DVI, NDRE, GNDVI, RVI, TCARI, GRVI, NDVI, and NBGVI
Accuracy_C	0.799	0.699	0.732
Precision_C	0.795	0.685	0.829
Recall_C	0.763	0.649	0.671
F1-score_C	0.779	0.667	0.699
Accuracy_V	0.791	0.821	0.860
Precision_V	0.789	0.786	0.850
Recall_V	0.850	0.846	0.850
F1-score_V	0.818	0.815	0.850

Notes: KNN: k-nearest neighbors; SVM: support vector machine; LR: logistic regression; C: calibration; V: validation.

**Table 6:** Basic statistics of the July dataset after outlier removal.

Vegetation Index	Mean ± SD	
	Noninfected (98)	Infected (99)
NDVI	0.881 ± 0.018 a	0.852 ± 0.024 b
GNDVI	0.776 ± 0.021 a	0.742 ± 0.025 b
GRVI	7.992 ± 0.791 a	6.836 ± 0.735 b
RVI	16.08 ± 2.296 a	12.86 ± 2.307 b
DVI	0.349 ± 0.036 a	0.297 ± 0.021 b
NDRE	0.472 ± 0.021 a	0.445 ± 0.027 b
NBGVI	−0.385 ± 0.022	−0.379 ± 0.026
OSAVI	0.726 ± 0.030 a	0.677 ± 0.027 b
TCARI	0.030 ± 0.031 b	0.070 ± 0.026 a

Notes: SD: standard deviation; a, b: significantly different (*p* < 0.05). The numbers in parentheses indicate the sample sizes after outlier removal.

**Table 7:** Classification results of each model for the July dataset.

Model	KNN	SVM	LR
Data split	7:3	8:2	8:2
Selected variables	TCARI, NBGVI, RVI, NDVI, GRVI, and GNDVI, NDRE	DVI, TCARI, NBGVI, NDVI, RVI, NDRE, GRVI, and GNDVI	DVI, NDVI, NBGVI, TCARI, RVI, NDRE, GRVI, and GNDVI
Accuracy_C	0.825	0.834	0.834
Precision_C	0.817	0.812	0.812
Recall_C	0.841	0.873	0.873
F1-score_C	0.829	0.841	0.841
Accuracy_V	0.917	0.925	0.925
Precision_V	0.903	0.870	0.870
Recall_V	0.933	1.000	1.000
F1-score_V	0.918	0.930	0.930

Notes: KNN: k-nearest neighbors; SVM: support vector machine; LR: logistic regression; C: calibration; V: validation.

**Table 8:** Basic statistics of combined dataset after outlier removal.

Vegetation Index	Mean ± SD	
	Noninfected (209)	Infected (195)
NDVI	0.896 ± 0.020 a	0.877 ± 0.031 b
GNDVI	0.786 ± 0.019 a	0.763 ± 0.029 b
GRVI	8.406 ± 0.759 a	7.552 ± 0.980 b
RVI	18.75 ± 3.232 a	16.24 ± 4.037 b
DVI	0.376 ± 0.038 a	0.338 ± 0.045 b
NDRE	0.476 ± 0.018 a	0.457 ± 0.025 b
NBGVI	−0.434 ± 0.052 b	−0.427 ± 0.053 a
OSAVI	0.751 ± 0.033 a	0.717 ± 0.046 b
TCARI	−0.019 ± 0.055 b	0.017 ± 0.061 a

Notes: SD: standard deviation; a, b: significantly different (*p* < 0.05). The numbers in parentheses indicate the sample sizes after outlier removal.

**Table 9:** Classification results of each model for the combined dataset.

Model	KNN	SVM	LR
Data split	8:2	7:3	8:2
Selected variables	OSAVI, DVI, NBGVI, NDRE, NDVI, and RVI	DVI, NDRE, NDVI, RVI, TCARI, and NBGVI	GRVI, GNDVI, DVI, NDRE, OSAVI, RVI, TCARI, and NBGVI
Accuracy_C	0.820	0.766	0.759
Precision_C	0.822	0.778	0.753
Recall_C	0.801	0.721	0.744
F1-score_C	0.812	0.748	0.748
Accuracy_V	0.815	0.754	0.778
Precision_V	0.833	0.764	0.784
Recall_V	0.769	0.712	0.744
F1-score_V	0.800	0.737	0.763

Notes: KNN: k-nearest neighbors; SVM: support vector machine; LR: logistic regression; C: calibration; V: validation.

## Data Availability

The data presented in this study are available on request from the corresponding author.

## Supplementary Materials

The following supporting information can be downloaded at: https://www.mdpi.com/article/10.3390/s26196119/s1, Figure S1: Spectral reflectance profiles of field-diagnosed and image-labeled infected pepper plants; Figure S2: Receiver operating characteristic (ROC) curves for the classification of field-diagnosed and image-labeled infected samples using the original five-band reflectance values; Figure S3: UAV imagery and selected spectral characteristics of pepper plants at different dates; Table S1: Comparison of five-band reflectance values between field-diagnosed and image-labeled infected samples; Table S2: Five-fold cross-validation classification performance for field-diagnosed and image-labeled infected samples based on five-band reflectance values; Table S3: VIF-based sensitivity analysis of the LR models for the June and July datasets; Table S4: Permutation feature importance of all variables selected for the best-performing models for the June, July, and combined datasets.

## Abbreviations

The following abbreviations are used in this manuscript:

AUC	Area under the curve
CM	Confusion matrix
DVI	Difference vegetation index
ExG	Excess green
FN	False negative
FP	False positive
GNDVI	Green normalized difference vegetation index
GSD	Ground sampling distance
GRVI	Green ratio vegetation index
KNN	k-nearest neighbors
LR	Logistic regression
ML	Machine learning
NBGVI	Normalized blue–green difference index
NDVI	Normalized difference vegetation index
NIR	Near infrared
NDRE	Normalized difference red edge
OSAVI	Optimized soil-adjusted vegetation index
PR	Precision–recall
RBF	Radial basis function
RE	Red edge
RGB	Red, green, and blue
ROC	Receiver operating characteristic
RVI	Ratio vegetation index
SD	Standard deviation
SVM	Support vector machine
TCARI	Transformed chlorophyll absorption in reflectance index
TP	True positive
TN	True negative
UAV	Unmanned aerial vehicle
