# Supplementary material for: Early Detection of Pepper Wilt Disease Using Unmanned Aerial Vehicle-Based Multispectral Time-Series Imagery
Source: Sensors (Basel). 2026 Sep 26;26(19):6119. doi: 10.3390/s26196119 (PMC13667695; doi:10.3390/s26196119)
Supplement: Supplementary file 1 [file sensors-26-06119-s001.zip › sensors-4565007-supplementary.pdf]

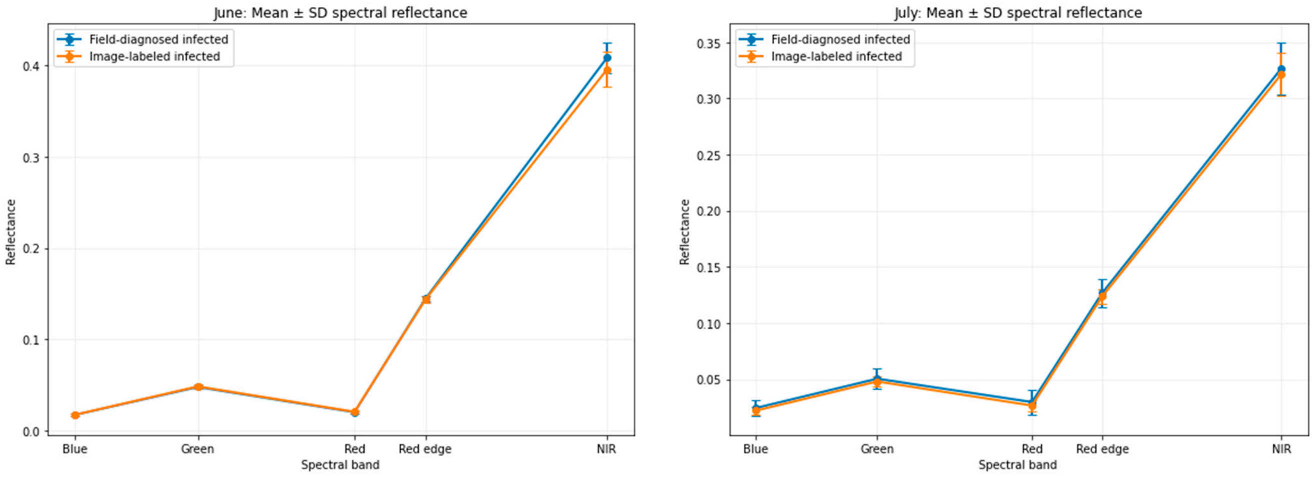

(**a**) (**b**)

Figure S1. Spectral reflectance profiles of field-diagnosed and image-labeled infected pepper plants: (a) June and (b) July. Points represent mean reflectance values, and error bars indicate standard deviations. SD, standard deviation; NIR, near infrared.

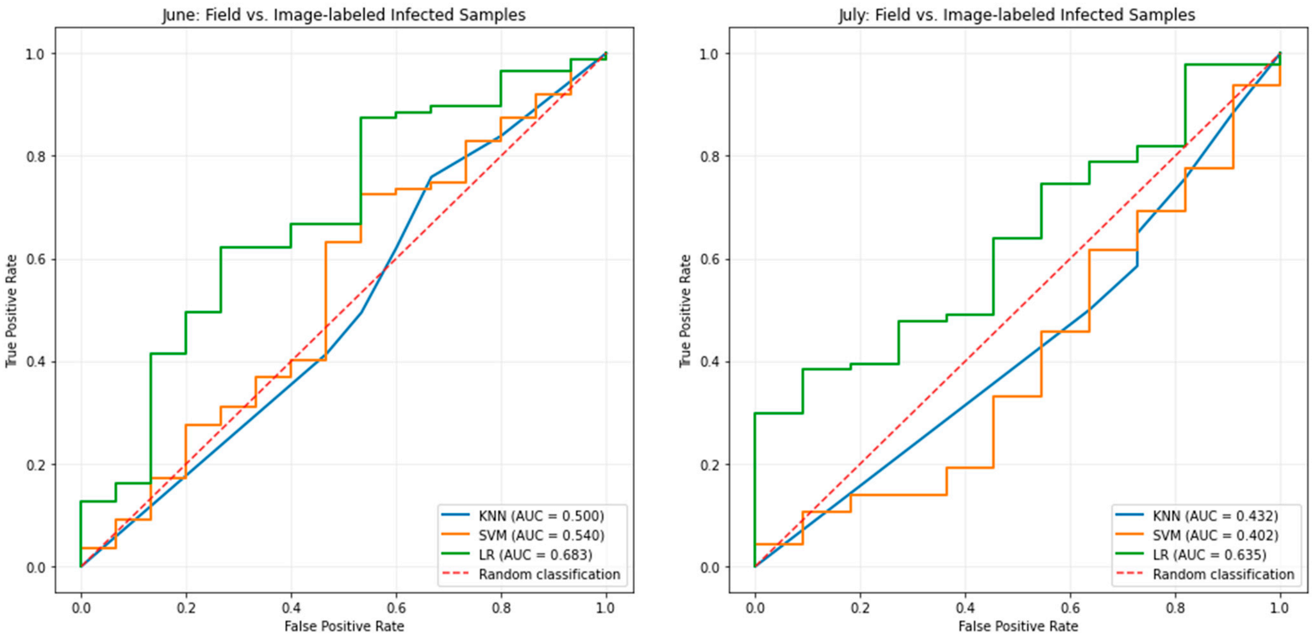

(**a**) (**b**)

Figure S2. Receiver operating characteristic (ROC) curves for the classification of field-diagnosed and image-labeled infected samples using the original five-band reflectance values: (a) June and (b) July. The KNN, SVM, and LR models were evaluated using stratified five-fold cross-validation with random oversampling applied only to the training folds. ROC curves and AUC values were calculated from the out-of-fold predictions pooled across the five folds. KNN, k-nearest neighbors; SVM, support vector machine; LR, logistic regression; AUC, area under the curve.

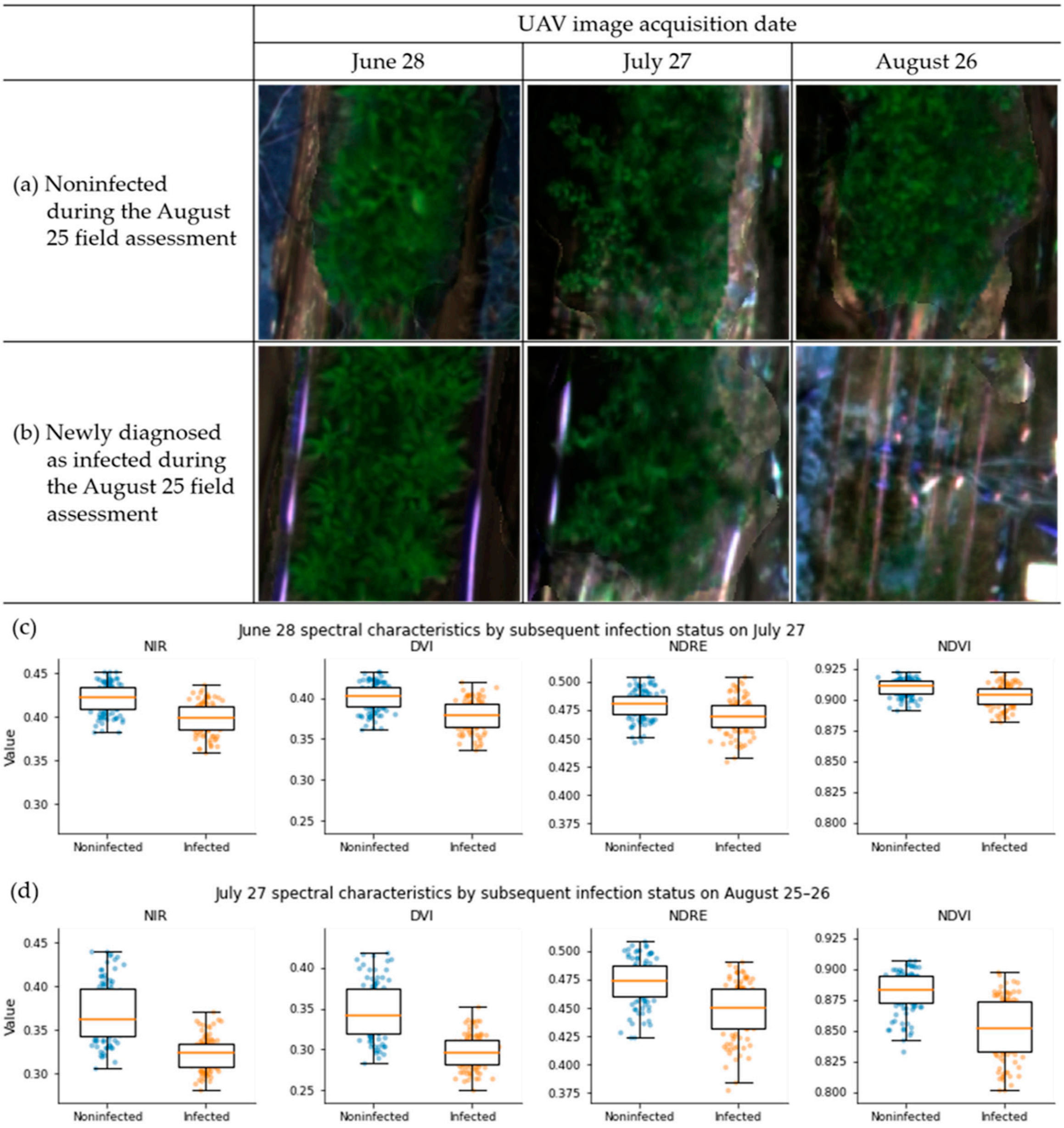

Figure S3. UAV imagery and selected spectral characteristics of pepper plants at different dates. (a) UAV imagery of a field-monitored plant that remained noninfected during the August 25 field assessment; the images were acquired on June 28, July 27, and August 26, 2023. (b) UAV imagery of a field-monitored plant newly diagnosed as infected during the August 25 field assessment, showing canopy presence in June and July followed by marked canopy loss in the August 26 image. Infection status was assessed in the field on August 25, whereas the corresponding UAV imagery was acquired on August 26. (c) Distributions of NIR reflectance, DVI, NDRE, and NDVI in the June 28 dataset according to the infection status subsequently determined on July 27. (d) Same distributions in the July 27 dataset according to the infection status subsequently determined on August 25–26. Individual points represent observations; boxes indicate the interquartile range with the median shown by the center line. UAV, unmanned aerial vehicle; NIR, near infrared; DVI, difference vegetation index; NDRE, normalized difference red edge; NDVI, normalized difference vegetation index.

Table S1. Comparison of five-band reflectance values between field-diagnosed and image-labeled infected samples. Values are presented as mean ± standard deviation. p-values were obtained using Welch's two-sample *t*-test.

Month	Band	Field-diagnosed reflectance value	Image-labeled reflectance value	p-value
June	Blue	0.017 ± 0.001	0.017 ± 0.001	0.865
	Green	0.048 ± 0.002	0.049 ± 0.002	0.223
	Red	0.020 ± 0.002	0.021 ± 0.002	0.328
	Red edge	0.145 ± 0.003	0.144 ± 0.003	0.319
	NIR	0.408 ± 0.017	0.395 ± 0.019	**0.014**
July	Blue	0.024 ± 0.007	0.022 ± 0.004	0.314
	Green	0.050 ± 0.009	0.048 ± 0.004	0.388
	Red	0.030 ± 0.011	0.026 ± 0.005	0.389
	Red edge	0.127 ± 0.013	0.124 ± 0.006	0.450
	NIR	0.327 ± 0.023	0.322 ± 0.019	0.490

Note: NIR: near-infrared. Boldface p-values indicate statistically significant differences at $p < 0.05$.

Table S2. Five-fold cross-validation classification performance for field-diagnosed and image-labeled infected samples based on five-band reflectance values. Values are presented as mean ± standard deviation across five folds.

Month	Model	Accuracy	Balanced Accuracy	ROC AUC
June	KNN	0.589 ± 0.051	0.511 ± 0.097	0.492 ± 0.207
	SVM	0.628 ± 0.100	0.589 ± 0.183	0.546 ± 0.135
	LR	0.638 ± 0.073	0.622 ± 0.109	0.681 ± 0.065
July	KNN	0.610 ± 0.085	0.458 ± 0.143	0.439 ± 0.227
	SVM	0.638 ± 0.170	0.517 ± 0.207	0.404 ± 0.167
	LR	0.714 ± 0.151	0.606 ± 0.093	0.669 ± 0.147

Note: KNN: k-nearest neighbors; SVM: support vector machine; LR: logistic regression; ROC AUC: area under the receiver operating characteristic curve. Random oversampling was applied only to the training data within each cross-validation fold to account for class imbalance.

Table S3. VIF-based sensitivity analysis of the LR models for the June and July datasets.

Dataset	VIF-reduced variables	VIF	Accuracy_V Original → Refined	Recall_V Original → Refined	F1-score_V Original → Refined
June	DVI; NDRE; TCARI; NBGVI	6.636; 3.585; 9.205; 2.907	0.860 → 0.837	0.850 → 0.800	0.850 → 0.821
July	DVI; NDRE; TCARI; NBGVI	7.202; 3.541; 7.941; 3.519	0.925 → 0.925	1.000 → 1.000	0.930 → 0.930

Note: VIF, variance inflation factor; LR, logistic regression; V, validation; DVI, difference vegetation index; NDRE, normalized difference red edge; TCARI, transformed chlorophyll absorption in reflectance index; NBGVI, normalized blue–green difference index. VIF values are presented in the same order as the listed variables. VIF was calculated using only the calibration subset of each monthly-dataset LR model with an 8:2 calibration-to-validation set ratio. Variables with VIF values of ≥10 were iteratively removed until all retained variables had VIF values of <10.

Table S4. Permutation feature importance of all variables selected for the best-performing models for the June, July, and combined datasets.

Dataset	Model	Variable	Permutation importance (Mean ± SD)
June	LR (8:2)	DVI	0.370 ± 0.069
		NDRE	0.100 ± 0.041
		GNDVI	0.088 ± 0.052
		RVI	0.073 ± 0.044
		TCARI	0.068 ± 0.046
		GRVI	0.039 ± 0.041
		NDVI	0.023 ± 0.030
		NBGVI	0.014 ± 0.021
July	LR (8:2)	DVI	0.240 ± 0.069
		NDVI	0.221 ± 0.066
		NBGVI	0.062 ± 0.038
		TCARI	0.060 ± 0.040
		RVI	0.045 ± 0.033
		NDRE	0.032 ± 0.035
		GRVI	0.019 ± 0.020
		GNDVI	0.003 ± 0.008
Combined	KNN (8:2)	OSAVI	0.179 ± 0.040
		DVI	0.162 ± 0.045
		NBGVI	0.161 ± 0.037
		NDRE	0.106 ± 0.039
		NDVI	0.104 ± 0.032
		RVI	0.096 ± 0.031

Note: Permutation feature importance was calculated on the validation dataset using classification accuracy as the scoring metric with 100 permutations. SD, standard deviation; LR, logistic regression; KNN, k-nearest neighbors; DVI, difference vegetation index; NDRE, normalized difference red edge; GNDVI, green normalized difference vegetation index; RVI, ratio vegetation index; TCARI, transformed chlorophyll absorption in reflectance index; GRVI, green ratio vegetation index; NDVI, normalized difference vegetation index; NBGVI, normalized blue–green difference index; OSAVI, optimized soil-adjusted vegetation index.
